# Methodological approaches for producing doubled haploids in sugar beet and red beet (Beta vulgaris L.)

**DOI:** 10.18699/VJ21.031

**Published:** 2021-05

**Authors:** T.R. Grigolava, A.V. Vishnyakova, A.A. Sinitsyna, A.V. Voronina, O.N. Zubko, O.V. Zudova, S.G. Monakhos

**Affiliations:** Russian State Agrarian University – Moscow Timiryazev Agricultural Academy, Moscow, Russia; Russian State Agrarian University – Moscow Timiryazev Agricultural Academy, Moscow, Russia; Russian State Agrarian University – Moscow Timiryazev Agricultural Academy, Moscow, Russia; Russian State Agrarian University – Moscow Timiryazev Agricultural Academy, Moscow, Russia; Russian State Agrarian University – Moscow Timiryazev Agricultural Academy, Moscow, Russia; Russian State Agrarian University – Moscow Timiryazev Agricultural Academy, Moscow, Russia; Russian State Agrarian University – Moscow Timiryazev Agricultural Academy, Moscow, Russia

**Keywords:** Beta vulgaris, haploid technology, gynogenesis, microspore culture, embryogenesis, doubled haploids, Beta vulgaris, гаплоидные технологии, гиногенез, культура микроспор, эмбриогенез, удвоенные гаплоиды.

## Abstract

The in vitro production of doubled haploids is a biotechnological path of an accelerated development of parental lines in F1-hybrid breeding programs. Unlike the traditional inbreeding method requiring 5 to 6 generations to reach a suf­f icient homozygosity of lines, the number of generations to produce pure lines of beet by haploid technologies is reduced to 2. The production of doubled haploids by gynogenesis is the most common biotechnological approach in sugar and red beets. Protocols for the production of doubled haploids for B. vulgaris species are few and have been developed mainly for sugar beets. There are no protocols for the production of doubled haploids for red beet (B. vulgaris convar. esculenta Salisb.), and the protocols developed for sugar beet (B. vulgaris convar. saccharifera Alef.) are ineffective for red beet, even though these two crops belong to the same species. The greatest success has been achieved in the production of doubled haploids by gynogenesis through isolated ovule culture, especially in sugar beet. Studies on the production of doubled haploids by androgenesis were actively carried out in the 1970s and 1980s and did not lead to the production of regenerated plants. However, at present, there is renewed interest among researchers in this approach, and scientists in different countries are conducting studies of Beta vulgaris androgenesis through isolated microspore culture. This article provides an overview of studies devoted to the production of doubled haploids, addressing the main problems of doubled haploid technologies, and methods to increase the frequency of embryogenesis and doubled haploid plant formation in B. vulgaris crops

## Abstract (ru)

Производство удвоенных гаплоидов in vitro – актуальный биотехнологический способ ускоренного создания родительских линий для селекции гибридов F1. В отличие от классического инбридинга время создания гомозиготных линий свеклы (Beta vulgaris) с помощью технологии удвоенных гаплоидов сокращается с пяти–шести до двух поколений. Гиногенез является наиболее распространенным биотехнологическим методом производства удвоенных гаплоидов сахарной и столовой свеклы. Протоколы производства удвоенных гаплоидов для видов B. vulgaris немногочисленны и разработаны в основном для сахарной свеклы (B. vulgaris convar. saccharifera Alef.). Наибольший успех достигнут в производстве удвоенных гаплоидов сахарной свеклы гиногенезом в культуре изолированных семязачатков. Для столовой свеклы (B. vulgaris convar. esculenta Salisb.) проведены единичные исследования с показанной низкой эффективностью производства гаплоидных растений андро- и гиногенезом. В итоге протоколы производства удвоенных гаплоидов столовой свеклы отсутствуют, а протоколы, разработанные для сахарной свеклы, неэффективны для столовой, несмотря на принадлежность к одному виду. Исследования производства удвоенных гаплоидов путем андрогенеза у представителей рода Beta активно проводились в 70–80-х гг. прошлого столетия и не закончились получением растений-регенерантов, однако в настоящее время среди ученых снова возник интерес к данному методу и в разных странах возобновлены работы по изучению андрогенеза у представителей рода Beta. Статья содержит обзор исследований, посвященных созданию удвоенных гаплоидов; обсуждение подходов решения основных проблем при получении удвоенных гаплоидов и методов, позволяющих повысить выход эмбриоидов и растений-регенерантов, а также удвоенных гаплоидов у растений вида B. vulgaris.

## Introduction

Subspecies of B. vulgaris is a valuable vegetable (red beet), fodder (feed beet) and technical (sugar beet) crops. Currently, the main trend in the breeding of these crops is development of F1 hybrids, based on the crossing of two homozygous parental lines. The traditional method to develop homozygous lines is inbreeding by self-pollination followed by selection of phenotypically uniform families over a minimum of 4–6 generations; this procedure takes 8–12 years for biennial crops (De La Fuente et al., 2013). The long-term development of parental lines by inbreeding is one of the limiting factors and a major problem in competitive F1 hybrids breeding. Doubled haploids (DH) technologies offer a time-saving approach to obtaining pure breeding lines by reducing the period to approximately 3–5 years (Zhuzhzhalova et al., 2020) and even less. A significant advantage of the doubled haploid production technology is the ability to achieve fully homozygous plant genotype in one generation. Another advantage of DH technology is the manifestation of recessive alleles in haploid plants masked in a heterozygous state in diploid plants, which facilitates the identification, assessment, and selection of plants with traits of agronomic importance (Doctrinal et al., 1989; Klimek-Chodacka, Baranski, 2013).

Doubled haploids of agricultural plants are produced in vivo by parthenogenesis or in vitro by isolated microspores culture, anthers, unfertilized ovules, etc. (Palmer, Keller, 2005). Among available DH technologies, the isolated unfertilized ovules culture (gynogenesis) is commonly used for doubled haploid production in B. vulgaris, particularly in sugar beet.

Sugar beet isolated unfertilized ovules culture is a simple but also a laborious technology. The frequency of embryos formation of the most responsive genotypes could reach 15 embryos per 100 cultivated ovules (Wremerth, Levall, 2003). Besides, the forming of mother plant clones from the somatic cells surrounding the embryo sac is not excluded. That makes it necessary to develop reliable methods for homo- and heterozygotes differentiation with the selection of the homozygotes among regenerated plants. Isolated microspores culture is a technology that avoids the culturing of somatic cells and somatic embryo formation. 

Isolated microspores culture technology and isolated anthers culture of beets was considered ineffective for a long time, and researchers managed to obtain only callus or proembryo structures without further regeneration, or somatic clones (Banba, Tanabe, 1972; Goska, Rogozinska, 1981; Van Geyt et al., 1985; Herrmann, Lux, 1988a). In 2017 Polish researchers managed to obtain embryos of red beet in microspores and anthers culture, however, the unrooted rosettes obtained from them died (Gorecka et al., 2017).

The purpose of this review is to collect and summarize data by various DH production approaches in B. vulgaris cultures, as well as outline the main problems and the ways to solve them.

## Historical overview of Beta DH technology development

The first haploids of sugar beet were discovered in 1945 by A. Levan (1945), later similar discoveries were reported by K. Zimmermann (1953), H.E. Fischer (1956), Th. Butterfass (1959), A. Kruse (1961) and B.L. Hammond (1966). Haploids were obtained from four types of material: 1) progeny of plants treated with polyploidizing agents; 2) offspring obtained from seeds of diploid or anisoploid varieties; 3) offspring obtained by vegetative propagation of diploid, cytoplasmically male sterile plants; 4) from anisoploid sugar beet plants.

Work on the experimental production of sugar beet haploids was started by N. Bosemark (1971): after pollinating sterile diploid beet plants with tetraploid plant pollen, he found about 0.2 % of haploids in the offspring. Attempts to produce haploids using distant hybridization were carried out in 1983 in Czechoslovakia: I. Seman crossed male sterile sugar beet plants with salad beets: the haploid yield was 0.013 % (Seman, 1983). A. Buchter-Larsen (1986) proposed an original method for the production of haploids – he combined pollination with irradiated pollen and the subsequent rescue of embryos, however, almost all plants turned out to be heterozygous for one or more genes. The obtained embryo rescue from pollination with pollen from other Beta species resulted in a relatively high yield of non-haploid plants (Buchter-Larsen, 1986). Since this method provided a low yield of haploids and was extremely laborious, the researchers abandoned attempts to produce doubled haploids in vivo.

Androgenesis is a simple and effective way of producing haploids in vitro in many crops. In the genus Beta, researchers have also attempted to produce haploids using isolated anther cultures. The first attempts to produce sugar beet haploids in vitro were undertaken by H. Banba and H. Tanabe (1972); when cultivating isolated anthers, they obtained one plant, the origin (somatic clone or haploid) of which is not indicated. M. Goska and J.H. Rogozinska (1981) continued their research on the cultivation of isolated sugar beet anthers and produced plants that were not androgenic.

D. Hosemans and D. Bossoutrot (1983) developed nutrient media for the cultivation of isolated ovules and carried out cytological studies of ovules developing on culture media. Their results showed that ovules containing a mature 7-nuclear embryo sac are most prone to in vitro development. The researchers obtained 17 haploid plants from 7237 isolated sugar beet ovules from male sterile donor plants.

Researchers have repeatedly attempted to obtain haploid beet plants by cultivating anthers or isolated microspores, however, they either failed to obtain full-fledged regenerant plants (Van Geyt et al., 1985; Gorecka et al., 2017; Gontarenko, Gerasimenko, 2018), or they were not haploids (Herrmann, Lux, 1988a). Therefore, gynogenesis became the main haploids producing method in sugar and red beets

The bulk of research on the study of gynogenesis was carried out on sugar beet. Researchers studied the influence on the frequency of embryogenesis and embryo germination/regeneration into a haploid plant of such factors as the type and concentration of growth regulators (D’Halluin, Keimer, 1986; Herrmann, Lux, 1988b; Ferrant, Bouharmont, 1994; Podvigina, 2003; Wremerth, Levall, 2003; Pazuki et al., 2018b), cold pretreatment of buds and ovules (Herrmann, Lux, 1988b; Svirshchevskaya, Dolezel, 2000; Podvigina, 2003; Pazuki et al., 2018b), shock stimulation by high temperatures of isolated ovules (Baranski, 1996; Wremerth, Levall, 2003), the time of ovule introduction into the culture in vitro (Lux et al., 1990; Baranski, 1996), the arrangement of buds on the inflorescence (D’Halluin, Keimer, 1986; Doctrinal et al., 1989; Podvigina, 2003), etc.

The gynogenesis of red beet is less studied. This topic was studied by R. Baranski (1996), who deliberated the effect of media growth regulators and the cultivation temperature of isolated ovules on the yield of embryos and callus, as well as the effect of the season of the year and mother plants growing condition (greenhouse/field) on the yield of regenerants. R. Baranski showed that the yield of regenerants is higher from the ovules taken from donor plants grown in the greenhouse compared to the field-grown donor plants. However, there was no significant difference in culturing efficiency of ovules obtained from donor plants grown in spring and summer seasons.

## Factors affecting DH production efficiency by isolated ovules culture

The process of haploid production is determined at the genetic level, but it is implemented depending on physiological conditions and inducing factors, which directly affects the frequency of haploid regenerants (Baranski, 1996; Podvigina, 2003). Major factors are the genotype of donor plant, the developmental stage of the female gametophyte, the location of the bud on the inflorescence.

Exogenous factors are of high importance and they affect the regenerative ability of cultivated ovules. These factors include season and duration of growing (age) of donor plants, cold and X-ray treatment of flower buds, the percentage ratio of growth regulators in culture media, temperature and other cultivation conditions of isolated ovules (Van Geyt et al., 1987; Lux et al., 1990; Gurel et al., 2000).

Among the factors influencing the embryogenesis efficiency, genotype is considered the most significant. Researchers pointed out that the most responsive to the inoculation of isolated ovules into the culture media are the hybrids and inbreed lines, but the CMS lines and varietypopulation have the lowest regenerative ability (Gurel et al., 2000; Podvigina, 2003)

In the genus Beta, a study of the number of genes, which controls the ability of genotypes in terms of forming embryos in vitro, was not carried out, probably, due to the low responsiveness of genotypes and also the technical laboriousness which is conjugated with haploids production. Responsive genotypes can be identified only experimentally. The responsive marker of sugar beet genotype may be the presence of abnormal structures in the male gametophyte (the presence of abnormal pollen grains and microspores) caused by dysfunction of the spindle apparatus, irregular formation of a callose wall, and the absence of cytokinesis during meiosis (Podvigina, 2003).

The selected buds location on inflorescence and the embryo sacs’ development stage are essentially important in sugar beet haploids induction. The highest regenerative activity is characterized by unfertilized ovules from buds 1 to 25 starting from bottom to top, the flower in the middle part of the inflorescence. In addition, the maximum yield of haploids is noted through the central shoot and first-order shoots compared to second-order branches (D’Halluin, Keimer, 1986; Doctrinal et al., 1989; Podvigina, 2003). The embryogenesis ability of isolated ovules is saved at all stages of the female gametophyte development; however, the 7th and 8th nuclear embryo sacs are the most responsive to embryogenesis and more easily pass from the gametophytic developmental pathway to the sporophyte one (Van Geyt et al., 1987; Podvigina, 2003). Markers of the 7th and 8th nuclear development stages of the ovule embryo sac for isolation and in vitro culture inoculation are the presence of mononuclear microspores and two-to three-core pollen in the anthers with ovules in one bud (Podvigina, 2003). Buds containing ovules at the appropriate stage of development can be found 1–5 days before flowering.

## Donor plants growing

Donor plants preparation is one of the most fundamental stages in doubled haploids production technology. Donor plants must be vigorous and healthy enough to produce high quality explants. W.E. Wremerth and M.W. Levall (2003) recommend adding solutions of macro- and microfertilizers weekly in order to grow healthy. Most researchers recommend growing donor plants in greenhouses or climatic chambers to minimize the impact of unfavorable weather factors and pest damage (Lux et al., 1990; Baranski, 1996; Gurel, 2000; Wremerth, Levall, 2003). However, in O.A. Podvigina studies (2003), the ovules which were taken from plants in the field had the highest regenerative capacity.

It is recommended to grow donor plants in summer; since the ovules from such plants are more responsive to in vitro cultivation compared to the ovules from those grown in autumn-winter season (Lux et al., 1990; Baranski, 1996). O.A. Podvigina (2003) made an interesting observation in terms of the relationship between the regenerative ability of ovules and climatic conditions during introduction period into culture – with sharp fluctuations in day-night air temperature, the yield of haploid seedlings has been increased.

## Embryogenesis induction

In B. vulgaris, heat treatment of buds and isolated ovules is used to stimulate embryogenesis: most often, the buds are pretreated at low temperatures 4–6 ° C for up to 5 days, followed by the cultivation of isolated ovules at 28–32 ° C (Lux et al., 1990; Baranski, 1996; Gurel et al., 2000; Podvigina, 2003; Wremerth, Levall, 2003). A. Pazuki et al. (2018b) showed that treatment of inflorescences for 7 days at 4 °C can stimulate embryogenesis in ovules; then, isolated ovules were cultivated at 27±2 °C in a climatic chamber with an 18-hour photoperiod. O.A. Podvigina (2003) directly cultivated isolated ovules of sugar beet at 4 °C for 5 days, which stimulated their development even on B5 hormone-free medium, however, the highest yield was observed on B5 medium supplemented with 2 mg/L gibberellin

O.A. Podvigina (2003) used ovules pretreated with X-rays to stimulate embryogenesis. Studies showed that the yield of haploid regenerants depended on the X-ray dose, the maximum frequency was 5.3 % at a treatment dose of 3000 roentgens, an increase in the radiation dose to 5000 roentgens did not have a stimulating effect and led to the appearance of unwanted mutations.

Many authors recommended isolated ovules cultivation at high temperature before the embryoids appearance (Lux et al., 1990; Baranski, 1996; Wremerth, Levall, 2003). However, there are also studies on lower temperature cultivation of isolated ovules (Baranski, 1996; Podvigina, 2003). O.A. Podvigina (2003) cultivated isolated sugar beet ovules at a temperature of 21–26 °C and showed that the optimum temperature is 23–25 °C. W.E. Wremerth and M.W. Levall (2003) developed a protocol to produce doubled sugar beet haploids, and according to the authors, the optimal temperature for isolated ovules cultivation is 30±2 °С; the maximum frequency of embryo formation in the most responsive genotypes was 15 %. R. Baranski (1996) conducted research on the influence of the temperature of isolated ovules cultivation in beet on the yield of embryoids. It was found that the temperature of 25 °C was the least favorable for development, and regenerant yield from the ovules was 4 %; there were no significant differences in regenerant yield between temperatures of 27 and 32 °C and the yield was 12.7 and 11.3 %, respectively

The isolated ovules are usually incubated in the dark until the appearance of embryoids/callus, after which they are placed in separate culture vessels and cultured in the light.

## Culture medium

The cultivation conditions of isolated ovules affect both the number of regenerants (embryoids and callus) and their quality. The correct selection of culture medium is important for the production of haploid plants in the culture of isolated ovules. 

Researchers use different nutrient media to cultivate isolated ovules, MS, N6, B5. The most commonly used solid media are MS and B5 with the addition of various growth regulators. Ovules were cultured on liquid media by H. Lux et al. (1990) and E.N. Vasilchenko et al. (2017). E.N. Vasilchenko et al., studying the effect of nutrition media concentration, showed that the proliferation of nuclei and cells of female gametophyte was activated on a liquid media, which had an effect on the initiation of neoplasms, and after transferring the resulting structures to solid media, the induction of haploid regenerants was observed.

Agar, agarose, and phytagel are usually used for culture media gelation in case of beet isolated ovules culture. Basically, for the culture of isolated ovules, media with the addition of agar or agarose are used (Baranski, 1996; Podvigina, 2003; Wremerth, Levall, 2003). W.E. Wremerth and M.W. Levall (2003) recommended using agarose as a gelling agent for embryo induction and doubled haploids production in sugar beet as well as agar in shoot and root induction media. In the studies (Gurel et al., 2000; Pazuki et al., 2017; Vasilchenko et al., 2017) was noted a positive effect of phytagel at a concentration of 2–3 g/L on embryogenesis and regeneration; its advantages are low consumption, low cost and impact similar to agar.

Growth regulators have the most significant influence on the development of explants (Seman, Farago, 1990; Gurel et al., 2000; Podvigina, 2003). During the culture, there are five pathways of development of unfertilized isolated ovules:

- Оne embryoid is formed from the ovule (direct regeneration).
- The ovule cells divide disorganized, resulting in the formation of callus tissue, from which secondary embryoids are formed.
- Degeneration of the primary regenerant into a callus-like structure and further secondary regeneration through the formation of adventive shoots.
- Formation of non-morphogenic callus.
- Formation of amorphous structures, transformation of the primary regenerant into a callus-like formation without further regeneration (Seman, Farago, 1990; Podvigina, 2003).

O.A. Podvigina (2003) and E.N. Vasilchenko et al. (2017) indicated that adding gibberellin (2 mg/L) to the culture medium causes embryogenesis, adding of auxins (IBA) and cytokinins (6-BAP and kinetin) to gibberellin stimulates the growth of callus along with embryoids and morphogenesis through all possible pathways of development of isolated ovules

W.E. Wremerth and M.W. Levall (2003) recommended using stepwise cultivation of isolated ovules on media with various combinations and concentrations of growth regulators. At the first stages of cultivation, media containing 2.4-D 0.5 mg/L and 6-BAP 0.3 mg/L are used in order to induce embryo or callusogenesis.

Sucrose is added to the medium as a source of carbohydrates. However, there is no consensus on the amount of sucrose in the culture medium, sucrose concentration varies within 30–100 g/L, depending on the technology. S. Gurel and his colleagues recommended adding sucrose to the medium for sugar beet embryogenesis at a concentration of 100 g/L (Gurel et al., 2000; Pazuki et al., 2018a, b). R. Baranski (1996) used culture media with the addition of 60 g/L sucrose to induce gynogenesis in red beet. W.E. Wremerth and M.W. Levall (2003) added 80 g/L sucrose to the embryo-induction medium and 20 g/L to the shoot-induction medium to produce sugar beet doubled haploids. H. Lux et al. (1990) added sucrose 100 g/L to the embryo induction medium and 20 g/L to the regeneration medium. The yield of embryoids varies greatly (widely) in each study, which makes it difficult to choose the optimal concentration of carbohydrates in the medium. However, in all studies, a general tendency is observed to induce embryogenesis, so that media with an increased content of carbohydrates are used, and for regeneration with a reduced one.

S. Gurel et al. (2000) have demonstrated that the addition of 0.5 % activated charcoal to the culture medium significantly increases the yield of embryoids (on average for genotypes from 3.3 to 12.8 %), and E.N. Vasilchenko et al. (2017) have indicated that the addition of 3 g/L of activated charcoal negatively affects the development of sugar beet regenerant plants, which may be due to the adsorption of hormones from the media. At the same time, E.N. Vasilchenko recommends the use of activated charcoal at the rooting stage, which can crucially increase the yield of rooted plants due to the adsorption of phenolic compounds that inhibit root formation.

While studying the induced embryogenesis of red beet, R. Baranski (1996) found that the highest yield of regenerants was observed on N6 medium (by Chu) when using a combination of 0.5 mg/L IAA and 0.2 mg/L 6-BAP; the yield of regenerants amounted to a maximum of 8.3 %.

## Plant regeneration

Рlant regeneration from embryos and/or callus is оne of the most significant stages in doubled haploids development. Researchers obtain regenerated plants either on the same culture medium on which ovules are cultivated (Baranski, 1996), or using media with different concentrations and growth regulators types (Podvigina, 2003; Wremerth, Levall, 2003; Vasilchenko et al., 2017).

O.A. Podvigina (2003) indicated that haploid plants at the first stages of development are characterized by poor development and viability, due to their haploid status. The death of plants at this stage can reach 45.5 %, depending on the genotype of the donor plant. To increase the yield of regenerant plants, it was proposed to introduce a stage of stabilization of haploids, including sequential plantregenerants cultivation on media without the addition of growth regulators, gibberellin, 6-BAP, and IBA. Several passages with alternating cultivation on media with growth regulators and without growth regulators made it possible to reduce the excess hormones concentration in the haploids tissues and stimulate them for further regeneration. It was also noted during the study that the ability of regenerated haploids to adapt to changing environment depends on the genetic characteristics of donor plants.

W.E. Wremerth and M.W. Levall (2003) have developed a technology of stepwise cultivation of sugar beet regenerants obtained from isolated ovules to solve the problem of viability of developing regenerant plants. For the shoot regeneration from sugar beet embryoids, it is recommended to use MS culture media containing kinetin (0.2 mg/L) and IAA (0.1 mg/L), the pre-root MS medium contains kinetin at a concentration of 0.5 mg/L, IBA at a concentration of 0.55 mg/L. For rooting it is recommended to use 1/2 MS medium with a high concentration of IBA (5.5 mg/L). A. Pazuki et al. (2018a) studied the effect of adding an anti-stress agent, the amino acid proline, on shoot and root formation in regenerated sugar beet plants obtained from isolated ovules. The addition of 0.2 or 0.3 mM proline to media stimulated active shoot formation and faster rooting of plants in comparison with the complete absence of proline or its concentration in the medium of 0.1 and 0.4 mM.

## Polyploidization

The cells of plants regenerating from ovules can be haploid, diploid, polyploid and occur in one regenerant in different proportions. The level of spontaneous diploidization in the studied accessions of sugar beet varies greatly: S. Gurel et al. (2000) recorded that only 5 % of plants underwent spontaneous diploidization; M. Goska (1997) obtained from 2 to 10 % of diploid sugar beet plants, and according to M. Tomaszewska-Sowa (2010), adding kinetin to the media gives diploid plants up to 93.8 %.

There are no reliable data on the factors that affect the degree of spontaneous diploidization of haploids; therefore, it is necessary to transfer the obtained haploid plants to the diploid level. Colchicine is usually used to double the chromosome number of haploid regenerant plants (Gurel et al., 2000; Podvigina, 2003). Haploid plants treatment is carried out by treating the meristematic tissues of a root crop or inflorescences with a solution of colchicine, by immersing the roots in a solution of colchicine or in a culture medium containing an antimitotic agent in vitro (Podvigina, 2003). Concentration and exposure may vary greatly: S. Gurel et al. (2000) recommended doubling the number of chromosomes by placing haploid plants on a medium supplemented with colchicine at a concentration of 5 g/L for 5 min. O.A. Podvigina (2003) pointed out 83.3 % the rate of diploidization when adding 0.05 % colchicine to the medium and exposure for 2 days.

Mixoploidy is a common phenomenon for sugar beet meristems (Kharechko-Savitskaya, 1940; Yudanova et al., 2004; Lukaszewska, Sliwinska, 2007); this complicates determining ploidy level of the developed plants.

The ploidy level of plants can be determined not only by direct counting of chromosomes number in the meristematic cells under microscope, but also by an indirect indicator – the number of chloroplasts in guard cells of the stomata (Yudanova et al., 2004). However, the chloroplasts number in stomatal guard cells may depend not only on the ploidy level, but also on the mode of reproduction (selfpollination or crossing). For example, in a diploid heterotic hybrid of sugar beet, the average number of chloroplasts can be 12–15 pcs. per cell, while in self-pollinated samples the average number of chloroplasts per cell is about 18, which makes this indirect method unreliable (Maletskiy et al., 2013).

The problem of determining the ploidy level of regenerated beet plants is solved by using flow cytometry (Gorecka et al., 2017; Vasilchenko et al., 2017) or absorption cytometry (Yudanova et al., 2004).

Some researchers (Podvigina, 2003; TomaszewskaSowa, 2010) considered it possible to distinguish haploid plants according to their phenotype: haploid plants are very different from diploid ones – they have small, numerous narrow leaves and a smaller habitus compared to diploids

## Microspores and anthers culture

Compared to gynogenesis, androgenesis is a less laborious method, because it does not require the isolation of small ovules. Isolated microspores culture allows to avoid formation of somatic clones as in the case of gynogenesis (from tissue surrounding embryo sac). In this connection, research in this area is promising, but in the genus Beta, such studies were rarely carried out and were not very successful.

Culture of isolated microspores and anthers culture are successfully used in many plant species to produce doubled haploids. However, haploid induction by isolated microspores and anthers in sugar beet and beetroot leads to the formation of proembryo structures, which sometimes form callus and/or roots. Early attempts to produce sugar beet haploids in anthers culture did not lead to obtaining androgenic plants (Banba, Tanabe, 1972; Goska, Rogozinska, 1981; Van Geyt et al., 1985; Herrmann, Lux, 1988a). One of the probable reasons for the failure of all researchers may be the presence of amyloplasts in pollen grains, which inhibits androgenesis due to the increased starch content in the plastids of mononuclear microspores (Sangwan, Sangwan-Norreel, 1987). The possibility of elimination starch grains in beetroot microspores was studied by K. Gorecka et al. (2017), for which two processing options were investigated. In the first case, donor plants were irrigated with a solution of gibberellin at a concentration of 50 mg/L, 250 ml per plant twice a week, which led to an increase in the yield of androgenic regenerants in only one of the accessions, while the other genotypes did not show any change in the yield of embryoids, and in one genotype there was no regeneration at all. In the second case, isolated anthers were kept in a solution of alpha-amylase at a concentration of 3 mg per 80 ml of water (from barley malt type VIII-A, Sigma-Aldrich) for 2 minutes, and then transferred to a medium for the induction of androgenesis, which led to the formation of 2 embryos in two genotypes. The researchers failed to obtain the plants, because regenerated rosettes turned black and died. M. Klimek-Chodacka and R. Baranski (2013) faced the same problem in some genotypes of beetroot in unfertilized ovules culture, which is connected with genotype-specificity.

K. Gorecka et al. (2017) obtained callus culturing isolated microspores and anthers in beetroot. The authors found that buds 1.3–1.5 mm long contain about 80 % of microspores of the mononuclear stage of development and about 15 % of the binuclear stage, which is the most optimal for the culture of isolated microspores and anthers in most crops. The authors indicated B5 with the addition of 100 g/L sucrose and 100 mg/L 2.4-D as the best culture medium for the cultivation of anthers and microspores. In the cytological examination of the obtained samples of callus and rosettes, the ploidy level was 4x, which indicates repeated endoreduplication in the callus tissue.

S.M. Gontarenko and G.M. Gerasimenko (2018) managed to obtain embryoids in the culture of isolated anthers in sugar beet; the embryoid yield was 0.15–0.92 %. They determined that the optimal stage of microspore development for the anther culture is the mononuclear stage. They showed that pretreatment of explants using low-temperature stress (4–8 °C) for 3–15 days is a factor initiating the transition of microspores from gametophytic to sporophytic development pathway, while pretreatment with high temperatures (30–32 °C) does not give positive effect. The best culture medium for the anther culture turned out to be half-concentration MS with the addition of a number of vitamins (B1 10 mg/L, B6 1 mg/L, PP 1 mg/L, C 1 mg/L) and amino acids (glutamic acid 250–500 mg/L, aspartic 30–50 mg/L, tyrosine 1–10 mg/L, arginine 2–10 mg/L, hydroxyproline 2–4 mg/L).

## Conclusion

The development of homozygous lines using haploid technologies remains in demand by breeders around the world. The production of pure lines using doubled haploids has several advantages over conventional methods: homozygosity is achieved in one generation, eliminating the need for several generations of self-pollination. The time saved is substantial, particularly in biennial crops. The most developed technology for DH lines production in beetroot nowadays is the technology of isolated ovules, which has been developed mainly on sugar beets. This technology for developing pure lines is rather laborious in comparison with the technology of isolated microspores culture. However, the latter is practically not applicable due to the lack of research and efficient protocol. In this regard, the study and development of the sugar and red beets doubled haploids technologies based on isolated microspores culture approach should be recommended.

## Conflict of interest

The authors declare no conflict of interest.
